# Enhanced Scytonemin Biosynthesis in Engineered *Nostoc flagelliforme* with ScyR Overexpression and Cultivation Optimization

**DOI:** 10.3390/microorganisms14102328

**Published:** 2026-10-13

**Authors:** Hao Xie, Yue Zhang, Xiaolong Yuan, Wanxia Xia, Xiang Gao

**Affiliations:** 1 School of Food and Liquor Engineering, Sichuan University of Science & Engineering, Yibin 644000, China; xiehaosg@suse.edu.cn (H.X.); xiaolongyuan407@suse.edu.cn (X.Y.); xiawanxia@suse.edu.cn (W.X.); 2 School of Food and Biological Engineering, Shaanxi University of Science & Technology, Xi’an 710021, China; zhangyue202503@163.com

**Keywords:** cyanobacteria, scytonemin, UV-protective pigment, transcriptional regulator

## Abstract

Scytonemin, a lipid-soluble indole-alkaloid pigment unique to cyanobacteria, is a bioactive small molecule with UV-shielding, antioxidant, and anti-inflammatory activities. *Nostoc flagelliforme* represents a promising strain for scytonemin biosynthesis, yet engineering to boost its yield remains challenging. Two genes encoding a histidine kinase (COO91_00772) and a response regulator (COO91_00771, ScyR) putatively constitute a two-component regulatory system governing the UV-induced biosynthesis of scytonemin. Here, we employed CRISPR/Cpf1 technology to achieve the insertion of an additional *ScyR* expression cassette into the *N. flagelliforme* genome and evaluated its effect on scytonemin yield. The engineered strain was designated Nf-psbA-ScyR, in which the *ScyR* gene was expressed under the P*psbA* promoter. This strain exhibited a markedly enhanced accumulation of scytonemin, reaching 4.6 mg g^−1^ dry weight under UV-B radiation after 15-day cultivation with exogenous tryptophan supplementation, representing a 1.9-fold increase compared with the *MAAs*-deficient (Nf-MAAs-D) strain. Upon transition from indoor UV-B exposure to outdoor solar radiation, scytonemin production further increased to 5.4 mg g^−1^ dry weight. These findings indicate that the ScyR regulator can highly promote scytonemin biosynthesis under UV induction, providing an engineering strategy for the exploitation of *N. flagelliforme*.

## 1. Introduction

Cyanobacteria are among the earliest photosynthetic organisms, having played an important role in the oxygenation of atmosphere during the early history of the Earth [1]. Beyond their fundamental contributions to oxygenic photosynthesis and carbon fixation, many cyanobacteria are diazotrophic and display remarkable morphological diversity, ranging from unicellular to filamentous and colonial forms. Their ecological versatility enables the colonization of freshwater, marine, and terrestrial habitats, where they serve as major drivers of global biogeochemical cycles. In these environments, cyanobacteria are continuously exposed to abiotic stressors, among which ultraviolet (UV) irradiation is particularly deleterious. UV exposure damages DNA, RNA, and proteins directly, or indirectly through the generation of reactive oxygen species (ROS) [2,3]. To mitigate UV-induced damage, cyanobacteria have evolved multifaceted protective strategies, including avoidance behaviors, activation of antioxidant systems, synthesis of UV-absorbing secondary metabolites, and energy-dependent repair and resynthesis of biomolecules [3].

*Nostoc flagelliforme*, a filamentous macroscopic cyanobacterium, naturally inhabits arid terrestrial environments, where it withstands recurrent desiccation and intense solar radiation. To cope with high UV exposure, *N. flagelliforme* has evolved intrinsic UV tolerance mechanisms [4], notably through the biosynthesis of scytonemin and mycosporine-like amino acids (MAAs), two classes of UV-absorbing metabolites [3,5,6]. MAAs are low-molecular-weight (less than 400 Da), colorless, and water-soluble compounds characterized by an aminocyclohexenone or aminocyclohexenimine core structure [7]. In contrast, scytonemin is a highly stable, yellow-brown, lipid-soluble indole-alkaloid pigment composed of a symmetrical dimer of fused heterocyclic units linked through an olefinic carbon atom [8]. Unlike energy-dependent repair systems, scytonemin confers constitutive photoprotection, remaining effective even during physiological dormancy such as desiccation, when metabolic activity is largely arrested [9]. Because scytonemin and MAAs provide broad-spectrum UV screening and antioxidant defense, these properties have stimulated growing interest in their exploitation for cosmeceutical and pharmaceutical applications. Thanks to its lipophilicity, scytonemin readily integrates into oil-based topical formulations, with bioactivities spanning photoprotection, anti-inflammatory effects and anti-proliferative effects, offering promise for biomedical applications [10,11].

**Figure 1:**
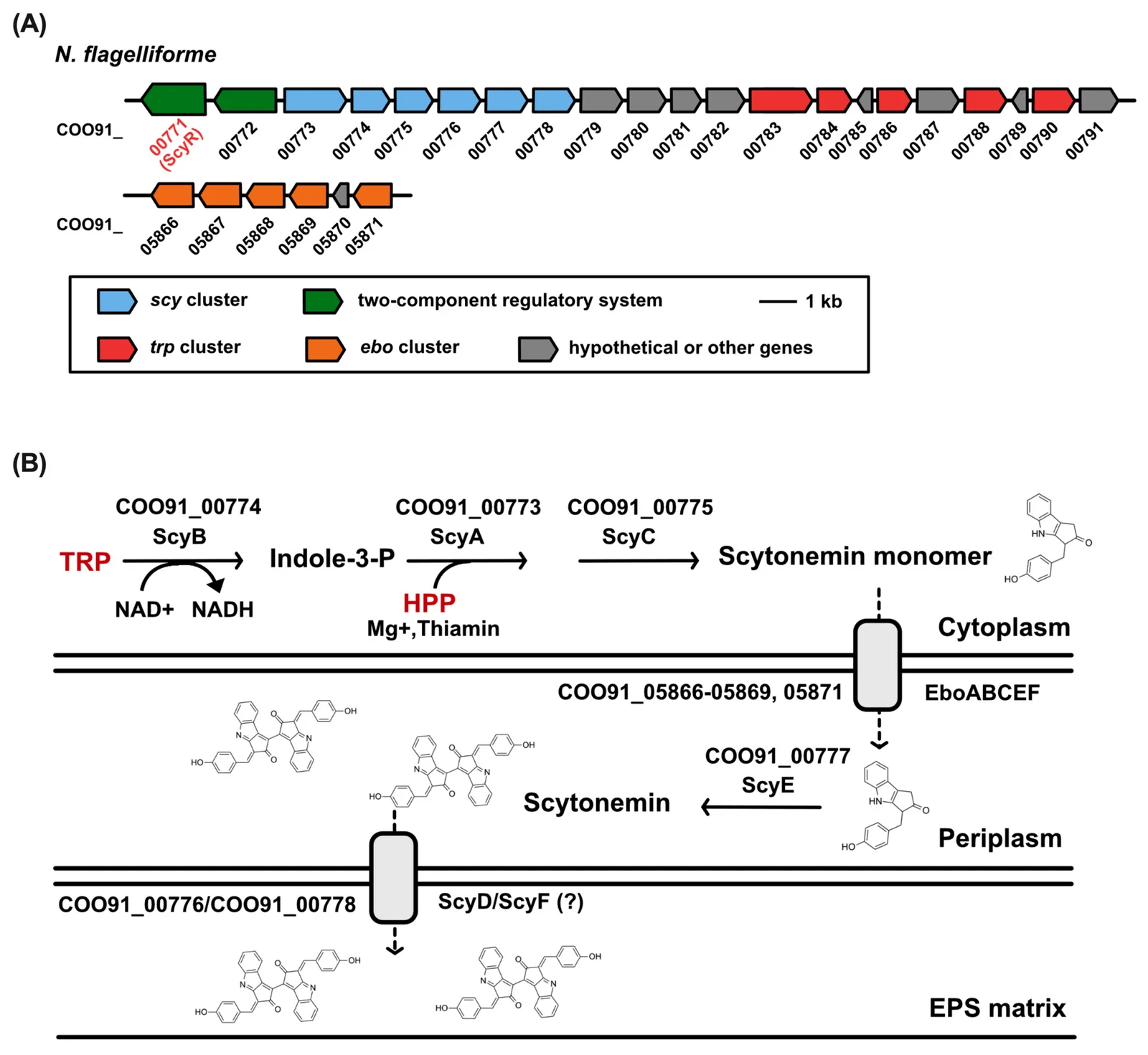
Scytonemin biosynthesis pathway and associated gene clusters in *Nostoc flagelliforme*. (**A**) Organization of scytonemin biosynthesis-associated gene clusters. (**B**) Schematic illustration of UV-induced scytonemin biosynthesis. EPS, exopolysaccharide; HPP, hydroxyphenylpyruvate; Indole-3-P, indole-3 pyruvic acid; TRP, tryptophan.

Scytonemin biosynthesis has been documented across diverse cyanobacterial genera, including *Nostoc* sp. [8], *Calothrix* sp. [12], *Scytonema* sp. [13], *Brasilonema* sp. [14], *Halothece* sp. [15], *Lyngbya* sp. [16], and *Anabaena* sp. [17], with the pigment localized within the extracellular polysaccharide sheath. The biosynthesis pathway was first elucidated in the cyanobacterium *Nostoc punctiforme* PCC 73102 through transposon mutagenesis [18], which identified an 18-gene cluster (*Npun_R1276* to *Npun_R1259*) essential for pigment formation. Comparative genomics revealed a homologous gene cluster in *N. flagelliforme* (Figure 1A) [4], while transcriptomic analyses demonstrated UV-B-induced upregulation of genes *COO91_00773-00778* and *COO91_00771-00772* [8].

The scytonemin gene cluster in *N. flagelliforme* comprises four functional modules: the *scy* genes (*scyA-F*) mediating pigment assembly from central carbon intermediates; a *trp* operon involved in precursor biosynthesis; an *ebo* operon facilitating monomer export to the periplasm; and a two-component regulatory system (encoded by *COO91_00771* and *COO91_00772*) that putatively modulates cluster expression. Within this pathway, ScyB, a leucine dehydrogenase homolog, initiates biosynthesis by converting L-tryptophan to indole-3 pyruvic acid (Figure 1B). ScyA subsequently catalyzes an acyloin condensation with *p*-hydroxyphenylpyruvate, followed by ScyC-mediated cyclization and decarboxylation to yield a ketone intermediate. This intermediate is oxidized to form the monomeric scytonemin precursor, which undergoes ScyE-catalyzed oxidative dimerization in the periplasm to produce the mature pigment, while ScyD and ScyF appear dispensable for pigment formation [19]. Based on genomic localization and structure similarity, the *COO91_00771* gene and its encoded protein *Nf*-ScyR are putatively functionally equivalent to the *Npun_F1278* gene and its encoded response regulator *Np*-ScyR from *Nostoc punctiforme* PCC 73102 [8,20]. Gene knockout experiments showed that *Np*-ScyR was essential for scytonemin biosynthesis in *N. punctiforme* PCC 73102 [20]. However, the extent to which either *Nf*-ScyR or *Np*-ScyR enhances scytonemin biosynthesis remains unknown.

Scytonemin formation relies on aromatic amino acid precursors derived from the shikimate pathway, where 3-dehydroquinate (DHQ) serves as a metabolic branch point for both MAAs and scytonemin biosynthesis [8]. We previously constructed an MAAs-deficient mutant (Nf-MAAs-D) [21]. In this study, we constructed the engineered strain Nf-psbA-ScyR via CRISPR/Cpf1-mediated genomic insertion at the *MAAs* gene cluster to investigate the effect of an additional *Nf*-*ScyR* expression cassette on scytonemin biosynthesis. Tryptophan serves as the direct substrate for scytonemin biosynthesis, and its supplementation can enhance scytonemin production [22]. Thus, we assessed scytonemin accumulation in three strains (WT, Nf-MAAs-D, and Nf-psbA-ScyR) under indoor artificial UV-B radiation with tryptophan supplementation. Further, the effect of outdoor solar irradiation was also examined. This research will highlight the regulatory effect of ScyR on scytonemin biosynthesis in *N. flagelliforme* and establish outdoor cultivation as a green strategy for scytonemin bioproduction.

## 2. Materials and Methods

### 2.1. Strains and Culture Conditions

*Escherichia coli* (*E. coli*) strain DH5α was used for plasmid construction and molecular cloning. *E. coli* strain HB101 harboring pRL443 and pRL623 [23] was used as the helper strain for conjugation with *N. flagelliforme* cells. Transformed *E. coli* cells were selected at 37 °C in LB medium supplemented with appropriate antibiotics. The cell suspension culture of *N. flagelliforme* was previously prepared in our lab [24]. *N. flagelliforme cultures* were routinely cultivated in BG11 medium in 250 mL Erlenmeyer flasks (ordinary glass) under 40 μmol photons m^−2^ s^−1^ at 25 °C, with horizontal shaking at 130 rpm.

### 2.2. Plasmid Construction

The plasmids constructed and used in this study are summarized in Table 1. The plasmid pHX1 was previously constructed and verified [21]. The plasmid pHX2 was generated using pHX0 [25] as the backbone. A spacer sequence was prepared by annealing complementary oligonucleotides (PAM-F/PAM-R) and ligated into the *Aar*I-digested plasmid pHX0 to generate the intermediate plasmid, pCpf1b-crRNA-Sp. The homologous recombination template consisted of two homology arms and the *COO91_00771* expression cassette. The homologous arms (L-HR and R-HR, each 800 bp) correspond to the 5′- and 3′-flanking regions of the *MAAs* gene cluster, respectively, and were amplified from *N. flagelliforme* genomic DNA using primer pairs L-HR-F/L-HR-R and R-HR-F/R-HR-R. Both homologous fragments were cloned into pCpf1b-crRNA-Sp at the *BamH*I site via In-Fusion assembly, generating pCpf1b-crRNA-HR-Sp. A *Sma*I site was introduced between L-HR and R-HR during assembly to facilitate subsequent cloning. The *COO91_00771* expression unit was constructed by fusing three fragments (P*psbA*, *COO91_00771*, and Kan^R^) via overlap extension PCR. The fragments P*psbA* and COO91_00771 were amplified from *N. flagelliforme* genomic DNA using primer pairs psbA-F/psbA-R and 00771-F/00771-R, respectively, while the fragment Kan^R^ was amplified from the plasmid pRL446 with the primer pair Kan-F/Kan-R. In addition, a 33-bp terminator sequence (5′-AAAATCAAAGGATCTTCTTGAGATCCTTTTTTT-3′) was inserted downstream of the *COO91_00771* coding sequence by incorporating the terminator as a 5′ extension of primer 00771-R. The assembled *COO91_00771* expression cassette was cloned into pCpf1b-crRNA-HR-Sp at the *Sma*I site to obtain the final plasmid pHX2. All constructs were verified by PCR amplification and Sanger sequencing. The oligonucleotides used in this study are listed in Table 2.

### 2.3. Generation of Engineered Strains via Conjugation

The plasmid pHX2 was transformed into wild-type *N. flagelliforme* cells via triparental conjugation [25] with slight modifications. Briefly, *N. flagelliforme* cells were grown in BG11 medium to mid-log phase (OD_750_ = 0.3–0.6). *E. coli* cells harboring cargo plasmid and *E. coli* HB101 conjugative cells harboring pRL443 and pRL623 were grown overnight at 37 °C. Ten milliliters of *E. coli* and *N. flagelliforme* cells were harvested by centrifugation (5000 rpm, 5 min) at room temperature. The *E. coli* cargo and conjugative cells were combined, washed twice with antibiotic-free LB medium, and resuspended in 1.5 mL of antibiotic-free LB. The *N. flagelliforme* cells were washed twice with fresh BG11 medium and resuspended in 1.5 mL of antibiotics-free BG11 medium. The *E. coli* mixture and *N. flagelliforme* cells were then combined and incubated at 25 °C for 1 h. After incubation, the mixture was spread onto sterile membranes placed on BG11 agar plates without antibiotics, followed by further incubation for 24 h. The membranes were subsequently transferred onto BG11 agar plates containing 30 μg mL^−1^ spectinomycin for transformant selection. Transformants were restreaked several times and cultured in liquid BG11 medium for plasmid curing, with the aid of Kan^R^ screening. Correct transformants of Nf-psbA-ScyR were verified by PCR using two pairs of primers (psbA-F/00771-R and PAM-F/R-HR-R), as well as by MAAs production analysis. The insertion at the *MAAs* gene cluster site was verified by extraction and detection of MAAs, as previously described [21,27], and no MAAs were detected in Nf-psbA-ScyR.

### 2.4. Cultivation and Scytonemin Induction

#### 2.4.1. The Effect of UV-B Intensity

Twenty mL of *N. flagelliforme* cell cultures were transferred into a 90 mm diameter quartz Petri dish. Cell cultures were exposed to UV-B radiation for 120 min. Two UV-B intensities (0.23 W m^−2^ and 0.56 W m^−2^) were applied; the dose was measured using a UV-radiation detector equipped with a UVB-X0 sensor (Shenzhen Linshang Technology Co., Ltd., Shenzhen, China). During the treatment, the maximum quantum yield of photosystem II (Fv/Fm) of the cell cultures was determined as previously described [28].

#### 2.4.2. Indoor Cultivation

Cell cultures were used to inoculate 100 mL of BG11 medium in 250 mL Erlenmeyer flasks (quartz glass) at an initial OD_750_ of 0.2–0.3. Cell cultures were irradiated with UV-B at 0.23 W m^−2^ for 1 h per day over 15 consecutive days. Samples (20 mL) were collected every 5 days for measurement of biomass, scytonemin, chlorophyll *a* (Chl *a*), carotenoids, and Fv/Fm value. The day 0 samples were collected during the preparation of the initial 100-mL cell cultures; thus, the remaining culture volume at the final sampling was approximately 60 mL. For tryptophan supplementation, 0.5 mM tryptophan was supplemented to BG11 medium at the beginning of cultivation.

#### 2.4.3. Outdoor Cultivation

Cell cultures were cultivated for most of the time in BG11 medium supplemented with 0.5 mM tryptophan under indoor conditions (25 °C, 40 μmol photons m^−2^ s^−1^, horizontal shaking at 130 rpm), except for a daily 1 h exposure to solar radiation. Each day, the cultures were transferred to an outdoor location on campus (34°22′43 N, 108° 58′7 E) and exposed to solar radiation for 1 h (12:00–13:00) over 8 consecutive days. Samples (15 mL) were collected every two days for the quantification of biomass, scytonemin, Chl *a*, and carotenoids. The day 0 samples were collected during the preparation of the initial 100-mL cell cultures; thus, the remaining culture volume at the final sampling was approximately 55 mL.

### 2.5. Growth and Pigment Determination

#### 2.5.1. Biomass Determination

Cell growth was assessed based on biomass accumulation. The sampled cultures were harvested by centrifugation at 7140× *g* for 10 min. The obtained cell pellets were freeze-dried using a vacuum lyophilizer, and the dry weight was recorded.

#### 2.5.2. Measurement of Fv/Fm

The maximum quantum yield of photosystem II (Fv/Fm) of cells was determined using a handheld fluorometer (AquaPen AP110/C, Photon Systems Instruments, Brno, Czech Republic) after 15 min of dark adaptation. Measurement settings were as follows: measuring light, 630 nm; flash pulse intensity, 60%; and super pulse intensity, 40%. Relative activity (%) was calculated as (B/A) × 100, where A and B represent the Fv/Fm values before and after radiation, respectively.

#### 2.5.3. Determination of Scytonemin, Chl *a*, and Carotenoids

Scytonemin, Chl *a*, and carotenoids were extracted from freeze-dried biomass. Briefly, dried cells were moistened and incubated in 4 mL of 100% acetone at 4 °C overnight. After centrifugation, the supernatant was filtered through a 0.22 μm organic membrane (PTFE membrane). The absorbance of a 2 mL aliquot was measured at 384, 490, and 663 nm, with 100% acetone serving as blank. Scytonemin, Chl *a*, and carotenoid concentrations were calculated according to the equations described by Fleming and Castenholz [29].

### 2.6. Statistical Analysis

Statistical analyses were performed using a two-way analysis of variance (ANOVA) followed by Tukey’s multiple comparisons test in Prism 8.0 (GraphPad Software, San Diego, CA, USA). Significance levels are indicated as * *p* < 0.05, ** *p* < 0.01, *** *p* < 0.001, and **** *p* < 0.0001.

## 3. Results and Discussion

### 3.1. Generation of Nf-psbA-ScyR Strain

**Figure 2:**
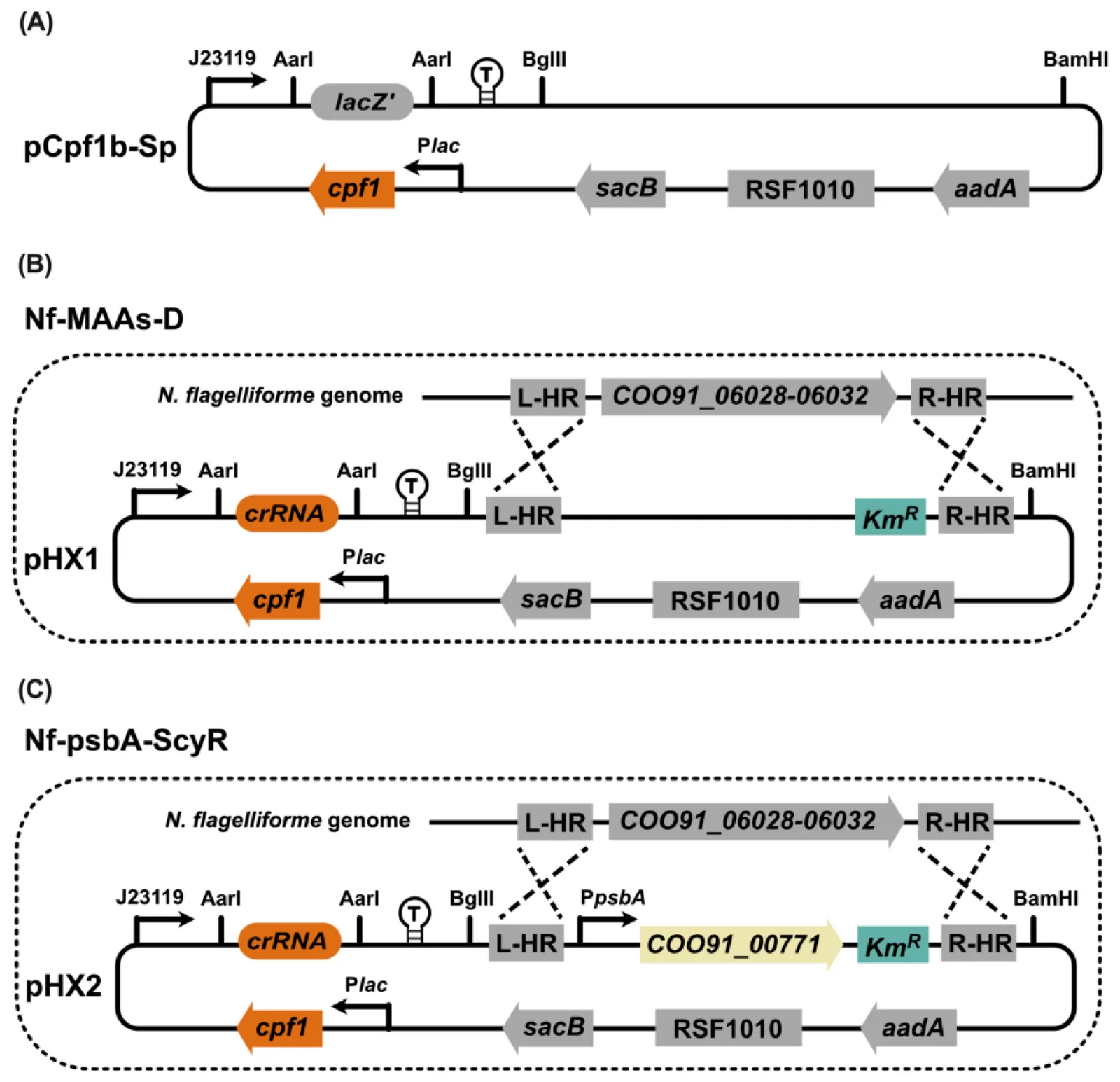
Schematic diagram of genetic constructs for engineering *N. flagelliforme*. (**A**) pCpf1b-Sp, the base vector for CRISPR/cpf1-assisted genetic modification. Expression of CRISPR RNA (crRNA) and *cpf1* was driven by promoter J23119 and P*lac*, respectively. (**B**) The plasmid pHX1 for construction of Nf-MAAs-D strain. The gene cluster *COO91_06028-06032* was replaced with a kanamycin resistance cassette (*Km^R^*) through homologous recombination [21]. (**C**) The plasmid pHX2 for the construction of the Nf-psbA-ScyR strain. The gene cluster *COO91_06028-06032* was replaced with the gene *COO91_00771* through homologous recombination. The expression of *COO91_00771* was driven by the native P*psbA* promoter. *aadA*, a spectinomycin resistance gene; *COO91_06028-06032*, the gene cluster responsible for MAAs biosynthesis; *cpf1*, a gene encoding the Cpf1 protein from *Francisella novicida*; *Km^R^*, kanamycin resistance gene; *lacZ’*, fragment encoding β-galactosidase LacZα for blue/white screening; *sacB*, a counter-selection gene conferring sucrose sensitivity; L-HR and R-HR, left and right homologous recombination regions flanking *COO91_06028-06032*.

*N. flagelliforme*, a non-model cyanobacterium, is recalcitrant to genetic manipulation via the pRL271-based homologous recombination method [30]. In contrast, the CRISPR/Cpf1 system has been shown to be effective [21] and was therefore adopted in this study to engineer *N. flagelliforme*. The plasmid pHX2 was constructed based on the pCpf1b-Sp vector and transformed into wild-type *N. flagelliforme*, generating the Nf-psbA-ScyR strain (Figure 2). Thus, the Nf-psbA-ScyR strain harbors an additional copy of the *COO91_00771* gene under the control of the native constitutive promoter P*psbA*, which was integrated at the *MAAs* gene cluster locus (Figure S1). The complete segregation of the Nf-psbA-ScyR strain was confirmed by PCR amplification (Figure S1). For comparative analysis, two other strains were included: wild-type *N. flagelliforme* (Nf-WT) and MAAs-deficient *N. flagelliforme* (Nf-MAAs-D). Nf-MAAs-D was constructed in a previous study (Figure 2B) [21]. As expected, neither of the engineered strains (Nf-MAAs-D and Nf-psbA-ScyR) produced MAAs due to the absence of the *MAAs* gene cluster following UV-B treatment (Figure S2). In contrast, Nf-WT produced MAAs in response to UV-B exposure, with production levels increasing with prolonged treatment. In the subsequent experiments, Nf-psbA-ScyR, as well as Nf-MAAs-D and Nf-WT, was used for comparative study.

### 3.2. Optimization of UV-B Intensity for Scytonemin Induction in N. flagelliforme

**Figure 3:**
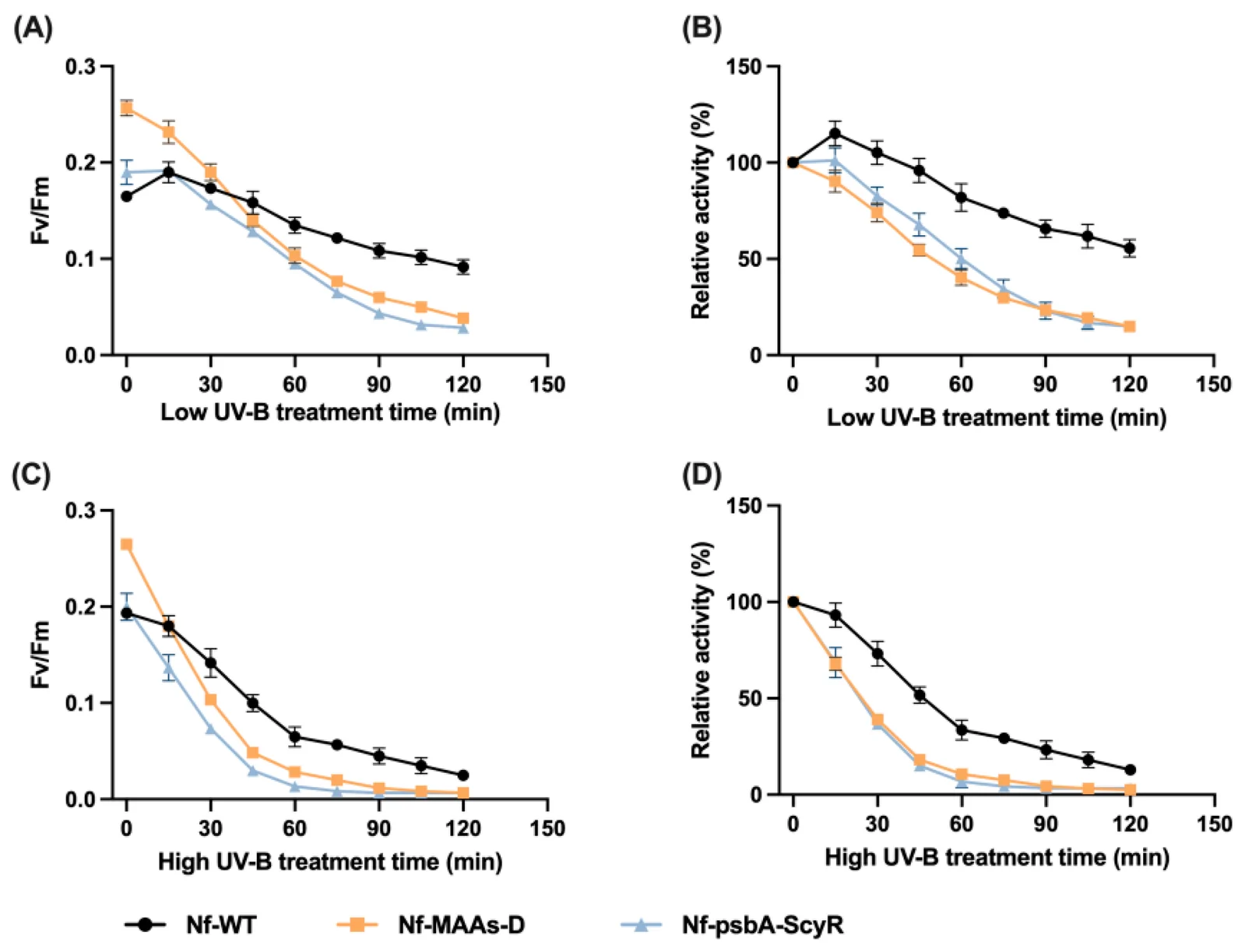
Photophysiological response of three *N. flagelliforme* strains (Nf-WT, Nf-MAAs-D, and Nf-psbA-ScyR) under low-dose (0.23 W m^−2^) or high-dose (0.56 W m^−2^) UV-B treatment. (**A**,**B**) Maximum quantum yield of PSII (Fv/Fm) and relative activity (%) of cells exposed to low-dose UV-B. (**C**,**D**) Fv/Fm and relative activity (%) of cells exposed to high-dose UV-B. Relative activity (%) was calculated as (B/A) × 100, where A and B represent the Fv/Fm values before and after radiation, respectively. Data represent the mean ± standard deviation (*n* = 6).

Scytonemin biosynthesis is known to be strongly induced by UV-B radiation [31]. Prior to the cultivation experiments, we determined the UV-B intensity and duration that effectively induced scytonemin biosynthesis while exerting only a moderate impact on growth, as indirectly indicated by the photophysiological parameter Fv/Fm. As described in a previous study [21], low-dose (0.23 W m^−2^) and high-dose (0.56 W m^−2^) UV-B were used to treat Nf-WT and Nf-MAAs-D strains. Under the same UV conditions, Nf-psbA-ScyR, together with Nf-MAAs-D, appeared more sensitive to the radiation than Nf-WT (Figure 3), underscoring the crucial role of MAAs in protecting cells against UV-B-induced damage [21]. High-dose UV-B had a severely inhibitory effect on cellular photophysiological activity during a 2 h exposure (Figure 3C,D and Figure S3). In contrast, low-dose UV-B irradiation impaired Fv/Fm to a lesser extent; after a 2 h exposure, Nf-psbA-ScyR still retained ~15% of its initial photophysiological activity (Figure 3A,B). Thus, low-dose UV-B irradiation was selected as the treatment condition for the subsequent experiments.

### 3.3. Optimization of Cultivation Conditions for Scytonemin Biosynthesis in N. flagelliforme

In a preliminary test, Nf-psbA-ScyR was cultivated for 12 h under low-dose UV-B radiation, with Nf-WT and Nf-MAAs-D as controls, but no detectable scytonemin accumulation was observed in any of the strains. We reasoned that continuous UV-B radiation may severely impair cell physiology and suppress scytonemin biosynthesis.

**Figure 4:**
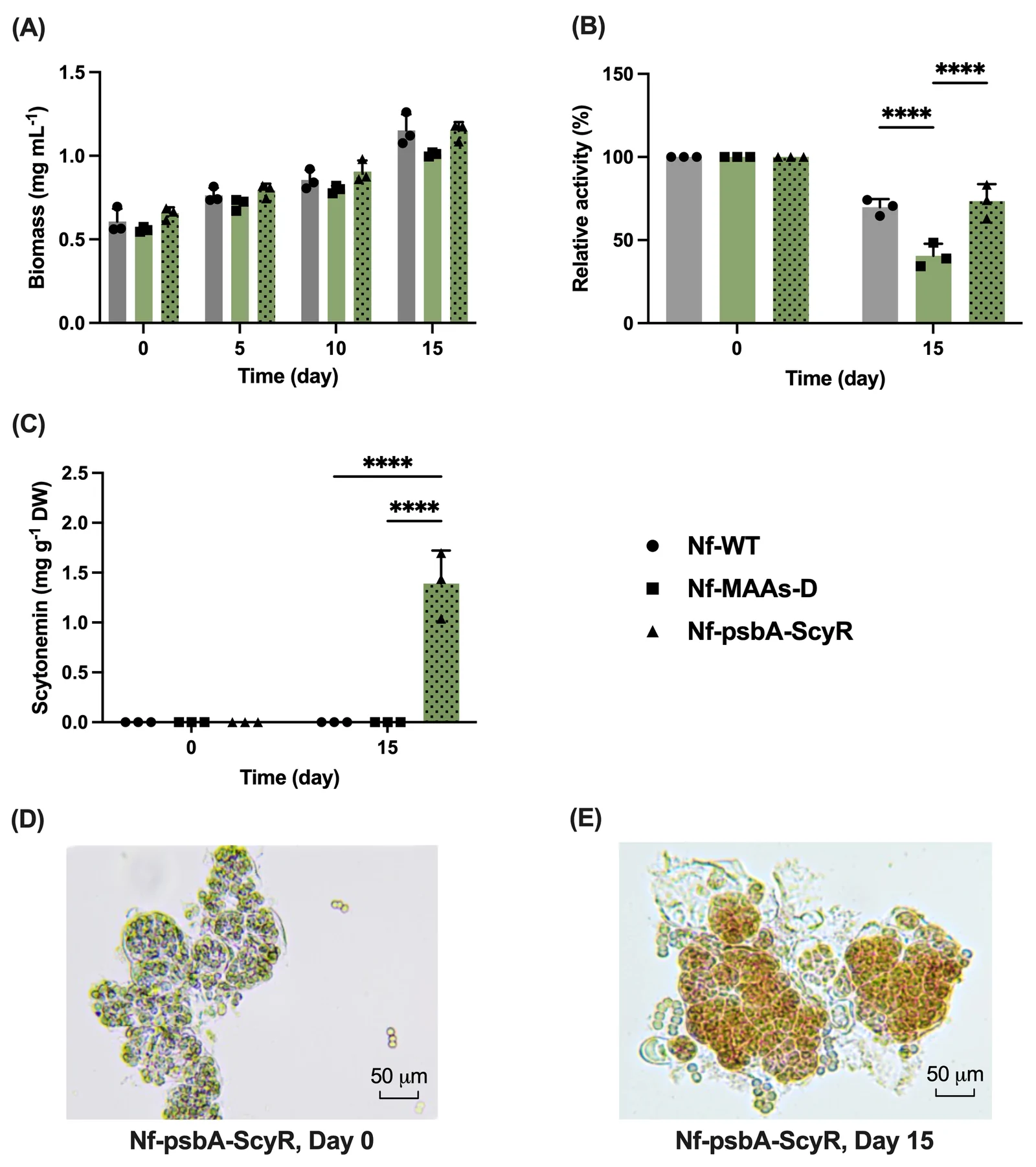
Growth, relative activity, scytonemin production, and pigmentation observations of Nf-psbA-ScyR under low-dose UV-B treatment. Nf-WT and Nf-MAAs-D served as controls. (**A**) Biomass accumulation in the three strains on days 0, 5, 10, and 15. (**B**) Relative activity (%) of three strains after a 15-day treatment. Relative activity (%) was calculated as (B/A) × 100, where A and B represent the Fv/Fm values before and after radiation, respectively. (**C**) Scytonemin production in the three strains on day 15. (**D**,**E**) Microscopic observation of Nf-psbA-ScyR colonies on day 0 and day 15, respectively. Data represent the mean ± standard deviation (*n* = 3); a two-way ANOVA was used to determine significance (**** *p* < 0.0001).

Following the periodic treatment strategy used previously [22], the cultivation period was extended to 15 days, with a daily 1 h exposure to UV-B radiation. During this period, the three tested strains exhibited increases in biomass, and no significant differences were observed among them (Figure 4A). On day 15, their photophysiological activities were still maintained but partially inhibited (Figure 4B). Nf-MAAs-D exhibited the greatest relative reduction in photophysiological activity. As shown in Figure 4C, neither Nf-WT nor Nf-MAAs-D accumulated detectable levels of scytonemin, indicating that intermittent low-dose irradiation was ineffective at inducing scytonemin in either strain; in contrast, remarkable scytonemin accumulation was detected in Nf-psbA-ScyR on day 15, reaching 1.4 mg g^−1^ DW. The presence of yellow-brown scytonemin in Nf-psbA-ScyR could be clearly observed within the surface layer of the cell sheath (Figure 4D,E). These results demonstrated the positive role of an additional *Nf*-*ScyR* expression cassette in promoting scytonemin production.

### 3.4. Positive Effects of Tryptophan Supplementation on Scytonemin Production

**Figure 5:**
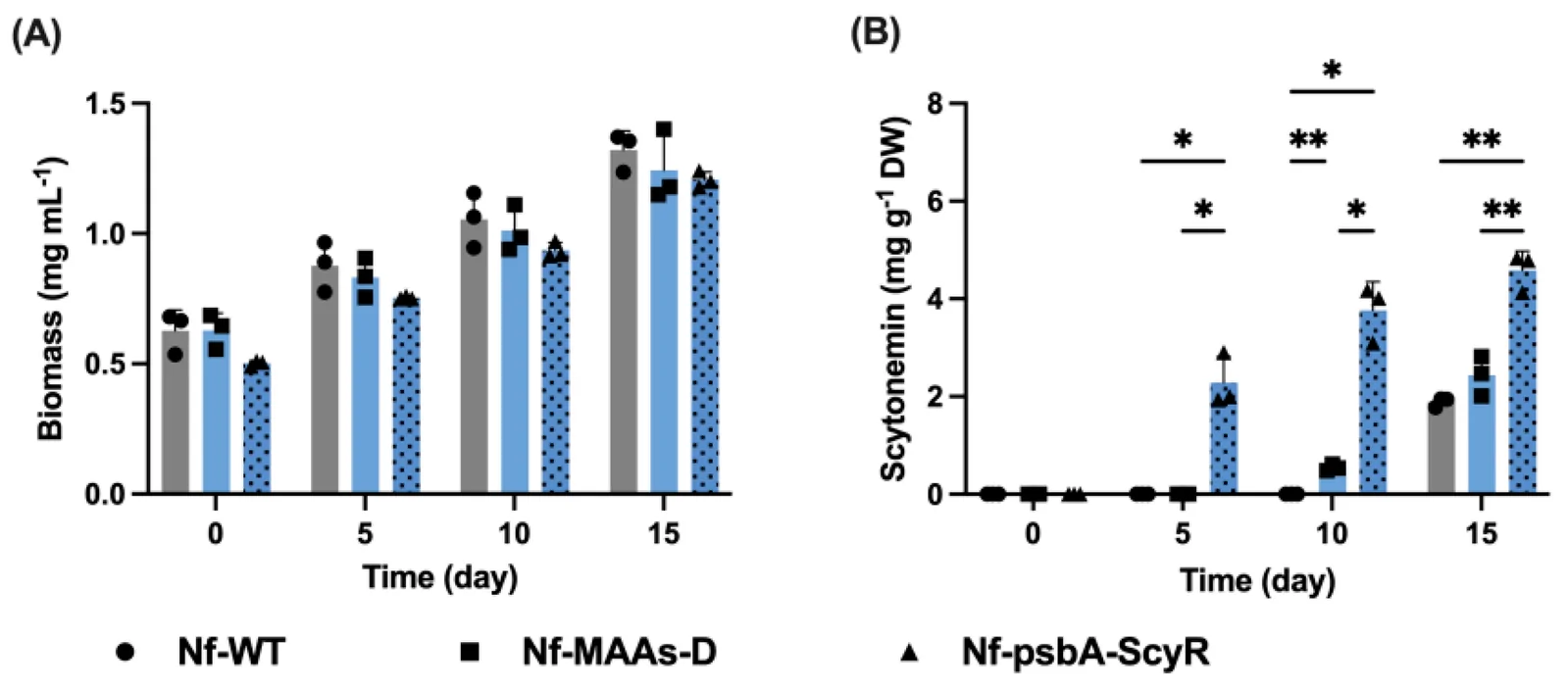
Biomass increase and scytonemin production of Nf-WT, Nf-MAAs-D, and Nf-psbA-ScyR under low-dose UV-B treatment with 0.5 mM tryptophan supplementation. (**A**) Biomass accumulation on days 0, 5, 10, and 15. (**B**) Scytonemin production on days 0, 5, 10, and 15. Data represent the mean ± standard deviation (*n* = 3); a two-way ANOVA was used to determine significance (* *p* < 0.05, ** *p* < 0.01).

Tryptophan serves as the direct substrate for scytonemin biosynthesis and has proven effective in enhancing scytonemin biosynthesis when exogenously added to the culture medium [22]. In this study, when 0.5 mM tryptophan was added, all three tested strains exhibited similar increase in biomass during the 15-day cultivation period (Figure 5A). The scytonemin production by the Nf-psbA-ScyR strain was increased by 3.3-fold, reaching 4.6 mg g^−1^ DW (Figure 5B), which was much higher than that obtained without tryptophan supplementation (Figure 4C). Notably, upon tryptophan addition, all three strains accumulated measurable amounts of scytonemin earlier (Figure 5B). On day 15, scytonemin production reached 2.4 mg g^−1^ DW and 1.9 mg g^−1^ DW for Nf-MAAs-D and Nf-WT, respectively, but was still much lower than that in Nf-psbA-ScyR. Thus, exogenous tryptophan supply exerted a marked additive effect in boosting scytonemin production in both engineered strains.

### 3.5. Induction of Scytonemin Biosynthesis by Outdoor Solar Radiation in N. flagelliforme

**Figure 6:**
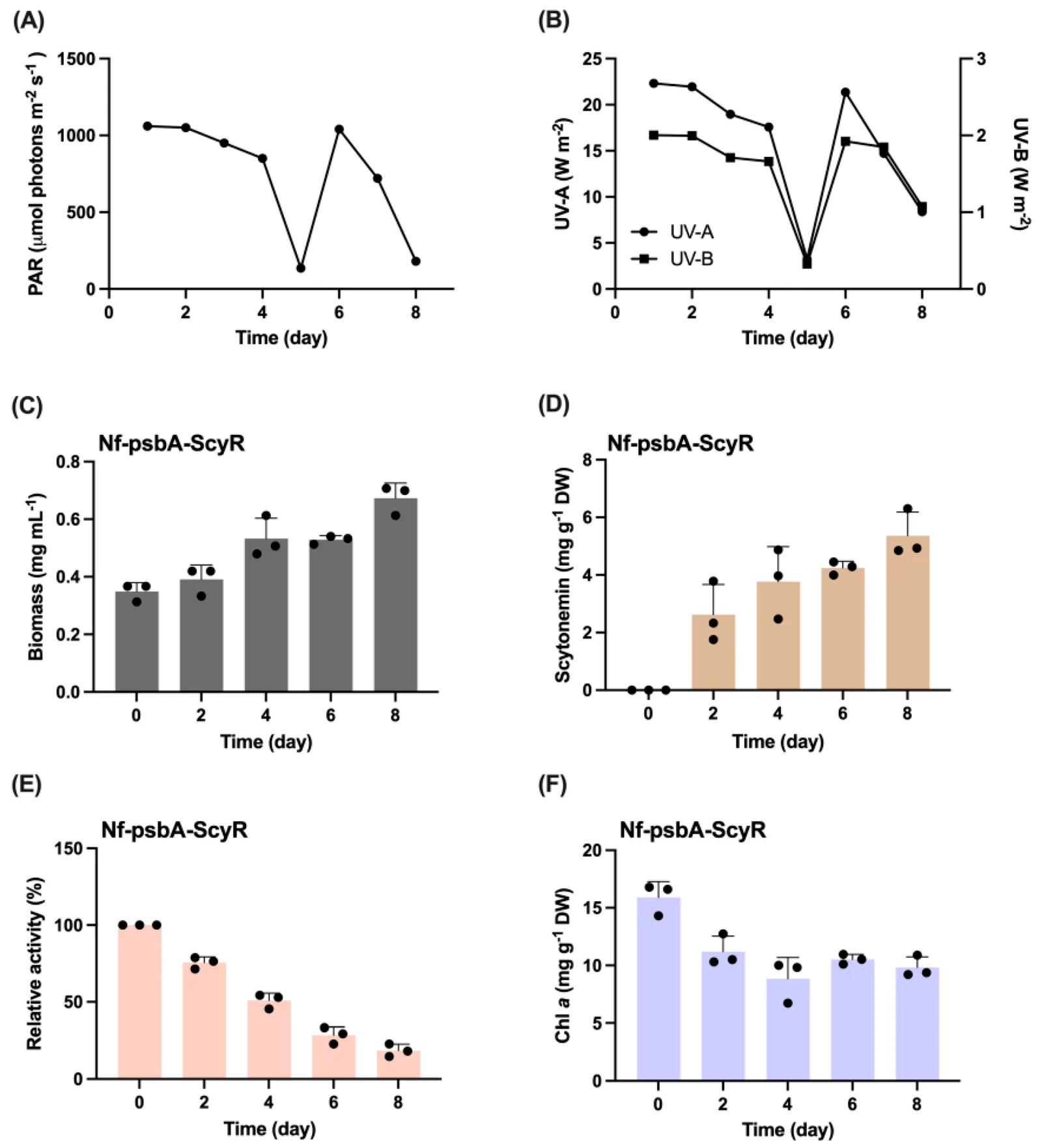
Time course of light intensities, biomass, and pigment concentrations of Nf-psbA-ScyR under solar radiation with 0.5 mM tryptophan supplementation. (**A**) Photosynthetically active radiation (PAR) intensity measured at 12:00 daily. (**B**) UV-A and UV-B radiation intensities measured at 12:00 daily. (**C**) Biomass accumulation of Nf-psbA-ScyR. (**D**) Scytonemin production of Nf-psbA-ScyR. (**E**) Relative activity (%) of Nf-psbA-ScyR. Relative activity (%) was calculated as (B/A) × 100, where A and B represent the Fv/Fm values before and after radiation, respectively. (**F**) Chl *a* content of Nf-psbA-ScyR. Data represent the mean ± standard deviation (*n* = 3).

Outdoor solar radiation is expected to provide a cost-effective approach to inducing scytonemin production in *N. flagelliforme*. During an 8-day cultivation period, cell cultures were moved outdoors for 1 h of daily exposure to solar radiation exposure to examine biomass, scytonemin production, and photophysiological state (Figure 6). The intensities of photosynthetically active radiation (PAR), UV-A, UV-B, and temperature were monitored (Figure 6A,B and Figure S4). During the cultivation period, the biomass of Nf-psbA-ScyR increased gradually (Figure 6C), whereas Chl *a* content declined from day 0 to day 2 and then stabilized at approximately 10 mg g^−1^ DW until the end of cultivation (Figure 6F). Photophysiological activity was gradually reduced (Figure 6E). Scytonemin was detected from day 2 and gradually increased over time, reaching a maximum of 5.4 mg g^−1^ DW on day 8 (Figure 6D). The major photosynthetic pigment Chl *a* and photophysiological activity decreased, whereas the UV-absorbing pigment scytonemin increased; and carotenoid levels were also less affected by UV exposure (Figure S5). This indicated the adaptive plasticity of Nf-psbA-ScyR in response to natural solar irradiation, which is a beneficial factor for outdoor cultivation and scytonemin production.

Replacing indoor UV-B radiation with natural solar radiation increased scytonemin production from 4.6 mg g^−1^ DW (achieved on day 15) to 5.4 mg g^−1^ DW (achieved on day 8; Figure 5B and Figure 6D). However, the photophysiological activity in Nf-psbA-ScyR decreased under prolonged solar irradiation (Figure 6E), which may restrict its cultivation over longer periods in terms of biomass accumulation and the efficiency of scytonemin biosynthesis. Two reasons may account for the higher efficiency of scytonemin production under solar radiation. First, natural solar radiation contains both UV-A and UV-B, which can synergistically activate scytonemin biosynthesis to a greater extent, thereby leading to higher accumulation. UV-A is more effective than UV-B at inducing scytonemin biosynthesis [8]. Second, the combined effects of high light intensity and temperature fluctuations under outdoor conditions may lead to oxidative stress, further promoting scytonemin synthesis as a protective response. Scytonemin possesses radical-scavenging activity, and its induction is potentially linked to the oxidative stress response [32]. It has been reported that high temperature and photooxidative conditions, in conjunction with UV-A radiation, increased scytonemin production in *Chroococcidiopsis* [33]. In addition, it should be noted that tryptophan supplementation is an efficient strategy for scytonemin biosynthesis, because de novo tryptophan synthesis from photosynthetic carbon fixation involves a long metabolic route in cyanobacterial cells [8].

Given that scytonemin accumulation is influenced by both cell growth and UV induction, a two-stage illumination strategy can be adopted to enhance scytonemin production. In the first stage, cultures could be grown under indoor PAR to maximize biomass accumulation. In the second stage, scytonemin synthesis could be induced by switching to outdoor solar radiation. It has recently been reported that attached-growth bioreactors are more effective than submerged-growth bioreactors for biomass accumulation and biofuel production [34,35]. Implementing this new cultivation technology may also help improve UV-induced scytonemin biosynthesis, as UV radiation can penetrate more effectively and enhance per-cell scytonemin accumulation.

## 4. Conclusions

In conclusion, this study evaluated the role of the response regulator ScyR (encoded by *COO91_00771*) as a positive modulator of scytonemin biosynthesis in *N. flagelliforme* under UV radiation. CRISPR/Cpf1-based genetic manipulation was employed to integrate an additional *ScyR* expression cassette in the *N. flagelliforme* genome. Indoor UV radiation and outdoor solar radiation were both effective at inducing scytonemin production, particularly when the medium was supplemented with exogenous tryptophan. The yield reached 4.6 mg g^−1^ dry weight after 15 days under indoor conditions, whereas it reached 5.4 mg g^−1^ dry weight after only 8 days under outdoor conditions, highlighting the potential of solar radiation as a green route for scytonemin production. It is also worth noting that tryptophan supplementation alleviated the metabolic bottleneck in substrate supply, enabling an earlier onset of scytonemin biosynthesis and higher yields. A limitation of this study is that, while the Nf-psbA-ScyR strain exhibited superior performance under outdoor conditions, it was evaluated only a limited number of times under the complex outdoor conditions. The optimal outdoor cultivation conditions and regimes still need to be explored. In addition, the integration of another expression cassette carrying the rate-limiting scytonemin biosynthesis genes into the Nf-psbA-ScyR strain would also be crucial for achieving high production of this high-value small molecule.

## 5. Patents

A patent has been granted based on the findings of this study (Patent No. ZL 202511075495.9, China). The funders had no role in study design, data collection and analysis, decision to publish, or preparation of the manuscript. The authors declare no other competing interests.

## Figures and Tables

**Table 1:** The plasmids and generated *N. flagelliforme* strains used in this study.

Designation	Description	References
**Strains**		
Nf-WT	wild-type *N. flagelliforme*	[26]
Nf-MAAs-D	*ΔCOO91_06028~06032*	[21]
Nf-psbA-ScyR	*ΔCOO91_06028~06032*::*COO91_00771*	This study
**Plasmids**		
pHX0	pCpf1b-Sp	[25]
pHX1	pCpf1-crRNA-HR(Km^R^)-Sp	[21]
pHX2	pCpf1-crRNA-HR(psbA-COO91_00771-Km^R^)-Sp	This study

**Table 2:** The oligos or primers used in this study.

Names	Oligos or Primers (5′-3′)
PAM-F	AGATGGCACTCTTGTATTACTAGCGA
PAM-R	AGACTCGCTAGTAATACAAGAGTGCC
L-HR-F	CGATATCTAGATCTCATGGAGTTACCGAGGTAGGTATG
L-HR-R	GTGAGATTTCGGTAATAATTCC
R-HR-F	ATTACCGAAATCTCACCCCGGGTACCAATCCACATTCTAAAGCC
R-HR-R	AACGTTGTTGCCATTGCGGAAGTGGTGAGTATGCTCAATG
psbA-F	GATCCCCACGGATCCGGGTTGTCATGTCACACATCCCC
psbA-R	GGTTTTATAAGTGCGGTTAG
00771-F	CGCACTTATAAAACCATGCATGAATCACCAAAGAAAAT
00771-R	AAAAAAAGGATCTCAAGAAGATCCTTTGATTTTTCAGCAATTTTGCCAAAGTTTTAC
Kan-F	GGGGAAAGCCACGTTGTGTC
Kan-R	CCCGGATCCGTGGGGATC

## Data Availability

The original contributions presented in this study are included in the article/Supplementary Material. Further inquiries can be directed to the corresponding author.

## Supplementary Materials

The following supporting information can be downloaded at https://www.mdpi.com/article/10.3390/microorganisms14102328/s1. Figure S1, Confirmation of insertion and complete segregation by PCR using genomic DNA templates from the Nf-WT and Nf-psbA-ScyR strains; Figure S2, Time course of mycosporine-like amino acid (MAA) production in three *N. flagelliforme* strains upon UV-B radiation; Figure S3, Changes in Fv/Fm values of cells before and after high-dose UV-B radiation; Figure S4, Time course of outdoor temperature when Nf-psbA-ScyR was exposed to solar radiation. Figure S5, Time course of carotenoid production in Nf-psbA-ScyR upon exposure to solar radiation.
