# Supplementary material for: Enhanced Scytonemin Biosynthesis in Engineered Nostoc flagelliforme with ScyR Overexpression and Cultivation Optimization
Source: Microorganisms. 2026 Oct 13;14(10):2328. doi: 10.3390/microorganisms14102328 (PMC13707963; doi:10.3390/microorganisms14102328)
Supplement: Supplementary file 1 [file microorganisms-14-02328-s001.zip › microorganisms-4566155-supplementary.pdf]

Supporting information for

Enhanced Scytonemin Biosynthesis in Engineered *Nostoc flagelliforme* with ScyR Overexpression and Cultivation Optimization

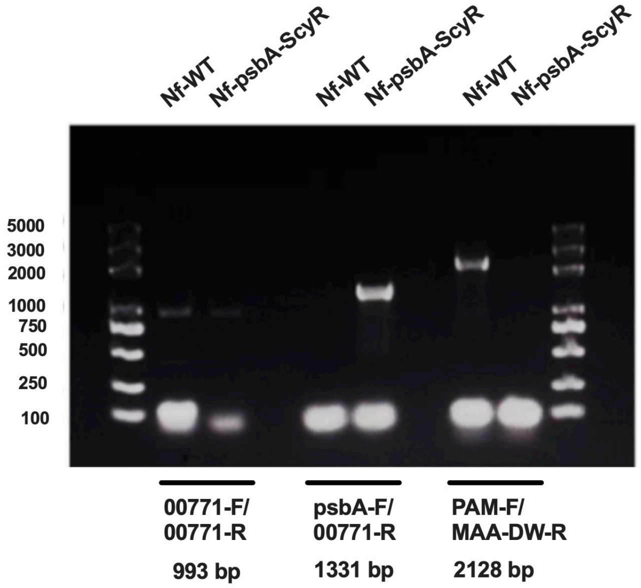

Figure S1 Confirmation of insertion and complete segregation by PCR using genomic DNA templates from Nf-WT and Nf-psbA-ScyR strains. Three pairs of primers were used: 00771-F/00771-R (expected size: 993 bp); psbA-F/00771-R (expected size: 1331 bp); PAM-F/MAA-DW-R (expected size: 2128 bp). The primer sequences are as follows: 00771-F (5′-CGCACTTATAAAACCATGCATGAATCACCAAAGAAAAT-3′), 00771-R (5′-AAAAAAAGGATCTCAAGAAGATCCTTTGATTTTTCAGCAATTTTGCCAAAGTTTTAC-3′), psbA-F (5′-GATCCCCACGGATCCGGGTTGTCATGTCACACATCCCC-3′), PAM-F (5′-AGATGGCACTCTTGTATTACTAGCGA-3′), and MAA-DW-R (5′-AACGTTGTTGCCATTGCG-GAAGTGGTGAGTATGCTCAATG-3′).

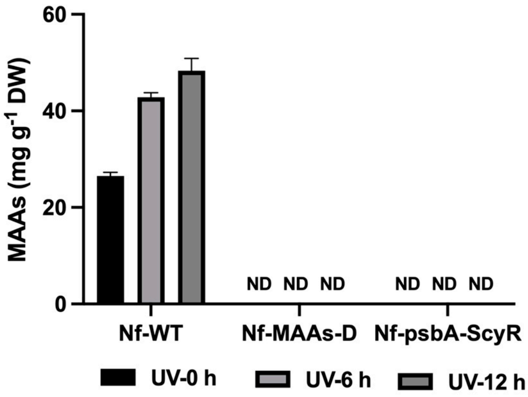

Figure S2 Time course of mycosporine-like amino acid (MAA) production in three *N. flagelliforme* strains upon UV-B radiation. Strains were were exposed to 0.8 W m^{-2} UV-B radiation for 0 h, 6 h, and 12 h. ND indicates that no MAAs production was detected. Data represent mean ± standard deviation (n=3).

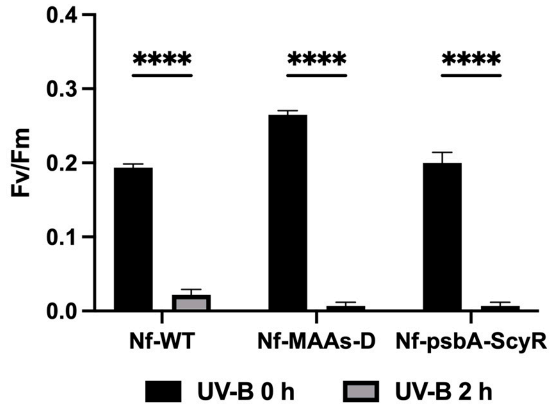

Figure S3 Changes in Fv/Fm values of cells before and after high-dose UV-B radiation. Cell culture were exposed to 0.56 W m^{-2} UV-B radiation for 2 h. Data represent mean ± standard deviation (n=6). **** indicates significance at $p < 0.0001$.

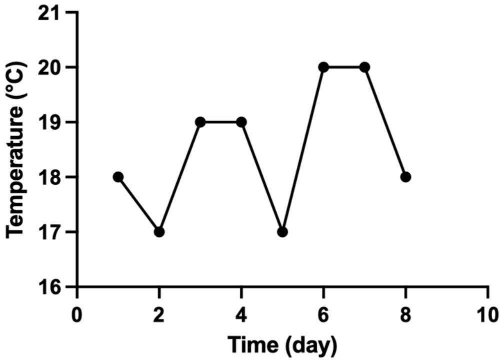

Figure S4 Time course of outdoor temperature when Nf-psbA-ScyR was exposed to solar radiation. During the 8-day cultivation, temperature was measured at 12:00 daily.

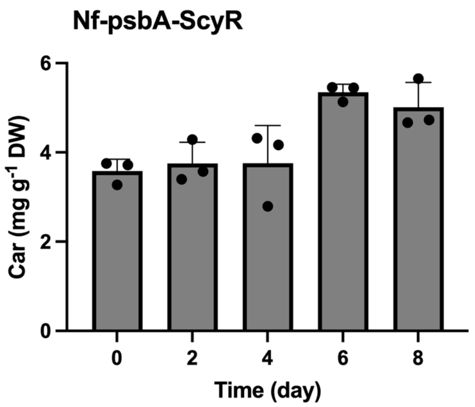

Figure S5 Time course of carotenoid production in Nf-psbA-ScyR upon exposure to solar radiation. The strain was cultivated in BG11 supplemented with 0.5 mM tryptophan and exposed to 1-h solar radiation daily over 8 days. Data represent mean ± standard deviation (n = 3). Car, carotenoids.
